# A late-diagnosed dermoid cyst complicated with a fistula in the left scapular region: a case report

**DOI:** 10.1093/jscr/rjac268

**Published:** 2022-06-24

**Authors:** Bana Zuhair Alafandi, Hiba Marstawi, Hiba Haj Saleh, Maria Chakhide, Aya Haji Mohamad, Aya Alayyoubi, Hasan Raslan, Aghyad Kudra Danial, Ahmad Al-Haj

**Affiliations:** Faculty of Medicine, University of Aleppo, Aleppo, Syrian Arab Republic; Faculty of Medicine, University of Aleppo, Aleppo, Syrian Arab Republic; Faculty of Medicine, University of Aleppo, Aleppo, Syrian Arab Republic; Faculty of Medicine, University of Aleppo, Aleppo, Syrian Arab Republic; Faculty of Medicine, University of Aleppo, Aleppo, Syrian Arab Republic; Faculty of Medicine, University of Aleppo, Aleppo, Syrian Arab Republic; Faculty of Medicine, University of Aleppo, Aleppo, Syrian Arab Republic; Department of Surgery, Aleppo University Hospital, Aleppo, Syria; Department of Surgery, Aleppo University Hospital, Aleppo, Syria

**Keywords:** dermoid cyst, fistula, scapular region, left shoulder, late-diagnosed, rare complication

## Abstract

Dermoid cysts are a rare case of developmental abnormality that results in benign tumors, which are classified into three categories based on their cause and appearance. Dermoid cysts tend to present within the first year after birth and are most commonly diagnosed by the age of 5. A 15-year-old girl presented with a complaint of localized, paroxysmal pain and malodorous fluid oozing from the left shoulder for the last 2 weeks. A fistulogram showed an extension of the fistula behind the clavicle and above the scapula with a cystic formation measuring ~2 cm on the upper part of the fistula tract, which called for appropriate surgical intervention. Our case is the first reported dermoid cyst in the left shoulder area associated with a fistula at birth, which is a rare complication since the complications of dermoid cysts differ depending on their location and size.

## INTRODUCTION

Dermoid cysts are a rare case of developmental abnormality that results in benign tumors. These tumors were classified into three categories upon cause and appearance: (i) congenital teratoma dermoid cysts; are mostly found in ovaries and testes; (ii) acquired dermoid cysts and (iii) congenital inclusion dermoid cysts [1]. A subdivision of the last category was created to study the correlation to the anatomical location of emergence, therefore, four subgroups were reported: first group cysts are located in the periorbital regions, which are the most common site for dermoid cysts; while the second group are the lesions overlying the dorsum of the nose, cysts found in the submental region and the floor of the mouth and the first and second branchial arches fusion areas in the midline in addition to lesions formed at the midventral and mid-dorsal fusion in the suprasternal, thyroidal and suboccipital regions fall into third and fourth groups, respectively [2]. Dermoid cysts tend to present within the first year after birth [3] but are most commonly diagnosed by age 5 [4]. Dermoid cysts represent 0.4–1.5% of all tumors [5], with an incidence rate of 3 in 10 000 children [4].

These cysts are typically asymptomatic, grow over time and usually expand through the cranium or spine [3, 4].

Complete surgical excision without ruptures is recommended to avoid complications and lower the risk of recurrence [4]. We, herein, present a case of a rare presentation of dermoid cysts in the left shoulder of a 15-year-old girl associated with a congenital draining fistula.

## CASE PRESENTATION

A 15-year-old girl was referred to the surgical clinic with a complaint of pain and malodorous fluid oozing from the left scapular region for the last 2 weeks.

The pain was localized, paroxysmal and relieved on analgesics. The patient had no significant medical history. There was no history of weight loss or loss of appetite, her menstrual cycle was normal, with no change in her bowel movement and her sleep was normal too and she had no other complaints.

**Figure 1:**
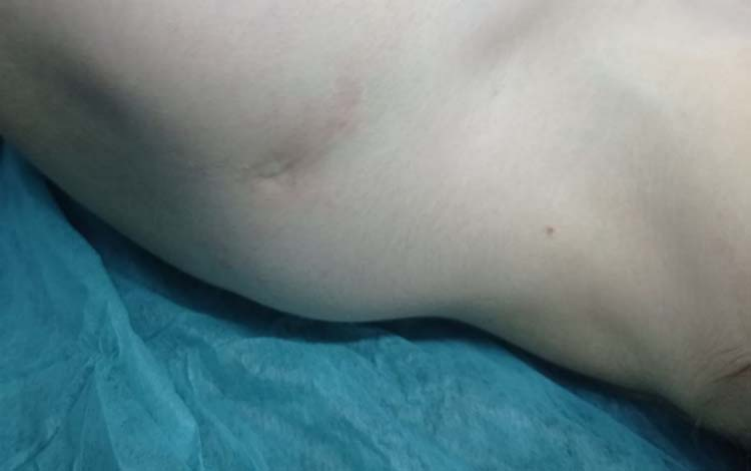
The dermoid cyst on physical examination.

Physical examination was normal, the patient was conscious and oriented with a good general condition. Chest examination revealed a fistula orifice that measured ~4 mm above and behind the left humeral head with redness around this orifice (Fig. 1), accompanied by pain but no reduced range of movement of the shoulder joint. A fistulogram showed an extension of the fistula behind the clavicle and above the scapula with a cystic formation measuring ~2 cm on the u-pper part of the fistula tract.

**Figure 2:**
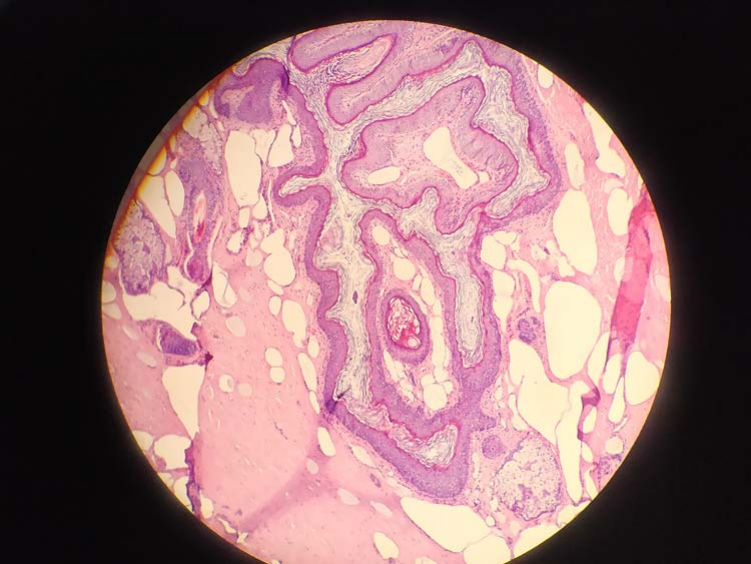
Biopsy confirms the diagnosis of dermoid cyst.

Surgical intervention was performed under general anesthesia to remove the cyst along with the fistula tract. Methylene blue was injected through the orifice and then both the cyst and the fistula were resected. Complete excision of the cyst and fistula tract was done, and a drainage tube was placed through the subcutaneous tissue. A biopsy confirmed the diagnosis of the dermoid cyst (Fig. 2).

Post-operative checks were within normal, fluid drained measured 5 mm and the patient was in good condition. The drainage tube was removed and the patient was discharged 24 hours post-operation in fine fettle. On follow-up, 15 days post-operation, the patient was in good health and her stitches were removed.

## DISCUSSION

Dermoid cysts are rare congenital developmental hamartomas usually arising from midline embryological defects from ectodermal trapping. Although dermoid cysts are mostly seen in the midline region of the head and neck [4], they can occur anywhere. Compared to a previous report of a similar case presenting on the right shoulder [6], our case is the first reported dermoid cyst in the left shoulder area associated with a fistula at birth.

Considering the congenital nature of these cysts, it is less likely for these tumors to present later in life; however, some cases documented in the literature reported the presentation of dermoid cysts through adulthood [4], which is in concordance with our case.

It is worth mentioning that there is no correlation between the gender or age of patients and the dermoid cyst location [4].

The complications of dermoid cysts differ depending on their location and the size to which it grows affecting surrounding tissues, since most dermoid cysts expand along the midline, they tend to enlarge and cause intracranial or intraspinal extensions along with craniofacial malformations [3, 7].

Torsion and wall disruption of the cysts are also common, leading to compression of the circulation and therefore infection and necrosis [5, 8].

One of the rare complications is the formation of a fistula; an abnormal tract most commonly seen between two internal organs, or between an internal organ and the body’s surface. Fistulas must be immediately diagnosed because they may hide deeper lesions, which coincides with our case [9]. Dermoid cysts are diagnosed when symptomatic [10], considering the size of the cyst that would trigger certain symptoms; however, in regard to our case, the presence of the fistula impeded the early diagnosis of the dermoid cyst, owing to the fact that once the cyst was enlarged enough to show symptoms, fluid accumulated within would leak through this tract, only to reduce the size of the tumor again. This resulted in this lesion’s late presentation and diagnosis until 15 years of age.

There have not been any reported cases of fistula formation due to dermoid cysts in this location, although fistula formation was reported in congenital teratoma dermoid cysts found in ovaries [11–13].

Dermoid cysts are benign tumors, but cases documented in the literature reported the occurrence of malignant tumors arising in ovarian dermoid cysts [2, 3, 14].

Typically, dermoid cysts are treated with complete surgical excision, and while small asymptomatic cysts do not require resection, it is common that dermoid cysts grow over time, necessitating surgery in order to avoid complications and lower risks of recurrence, as was the case of our patient [4].

As the first reported case of a congenital fistula due to a dermoid cyst in the shoulder, we believe that further identification of cases misdiagnosed at an earlier age is required to identify the prevalence of such tumors and update on the associated anomalies and management of these lesions to better assess all aspects of such developmental defects.
